# Ceftriaxone-induced hemolytic anemia managed successfully in a 54-year-old woman: a case report and literature review

**DOI:** 10.3389/fphar.2024.1505366

**Published:** 2025-01-07

**Authors:** Liqian Zhang, Wenfeng Huang, Jiakai Xu, Yunxing Li, Jihong Zhu

**Affiliations:** Department of Emergency, Peking University People’s Hospital, Beijing, China

**Keywords:** ceftriaxone, hemolytic anemia, drug-induced immune hemolytic anemia, complications, treatment

## Abstract

Ceftriaxone is widely used in clinical practice for its efficacy against infections. However, its increasing association with life-threatening immune hemolytic reactions urge clinicians to enhance recognition and maintain sharp vigilance. This report details a rare and severe case of ceftriaxone-induced hemolytic anemia (CIHA), hemodynamic instability and hemolytic crisis in a 54-year-old woman after intravenous infusion of ceftriaxone following a respiratory infection. Clinicians must promptly identify symptoms suggestive of CIHA, such as fatigue, pallor, nausea, vomiting, and trunk pain, and immediately discontinue ceftriaxone. Laboratory examination can also assist in confirming the diagnosis of CIHA. Effective management measures include rigorous monitoring of vital signs, circulatory support, respiratory support, timely blood transfusion, administration of steroid hormones, IVIG infusion as necessary, plasma exchange, and symptomatic treatment of possible complications. Even after the patient has achieved full recovery, careful consideration should be given to the choice of subsequent antibiotics to prevent recurrence of CIHA.

## Introduction

Ceftriaxone (CTX), a third-generation cephalosporin antibiotic, is widely utilized due to its broad-spectrum antibacterial activity, convenient administration, adjustable dosage, and limited cross-reactivity with other drugs. Although clinical adverse reactions caused by ceftriaxone are relatively uncommon and generally mild, there is potential for serious adverse reactions, including liver injury, neurological adverse events, shock, and hemolytic anemia, which may even pose a serious threat to the lives of patients (Rolain et al., 2024; Tao et al., 2024; Cannavino et al., 2016; Guarino et al., 2022).

Drug-induced immune hemolytic anemia (DIIHA) is an exceedingly rare condition, with a reported incidence of approximately 1 in 1 million individuals (Garratty, 2009). Its pathogenesis is intricate, primarily involving immune mechanisms mediated by drug-dependent or non-drug-dependent antibodies (Arndt, 2014). The current diagnosis of DIIHA relies mainly on laboratory tests, with patients typically presenting hemolytic manifestations such as decreased hemoglobin levels and hyperbilirubinemia, along with positive direct anti-human globulin tests (DAT) for anti-C3 and/or IgG/IgM (Arndt, 2014; Salama and Mayer, 2014). Previous research has indicated that ceftriaxone-induced hemolytic anemia (CIHA) is among the more common clinical types of DIIHA, commonly associated with severe complications including multiple organ failure and a high fatality rate ranging from 20% to 50% (Dicaro et al., 2024; Neuman et al., 2014; Renard and Rosselet, 2017). Given the widespread use of ceftriaxone in clinical practice, timely identification and appropriate management of CIHA are imperative.

This report presents a case of a 54-year-old patient with CIHA, and provides an in-depth review of the associated pathogenesis, diagnosis, and treatment options for CIHA, aiming to enhance clinicians’ awareness of this rare and potentially fatal disease.

## Case description

A 54-year-old woman with a medical history of type 2 diabetes, hypertension, and bronchial asthma was transferred to our hospital with complaints of sore throat, cough lasting for 10 days, intermittent fever, and dark-colored urine for the past 9 days. The patient presented with a sore throat and cough 10 days ago, without an identifiable cause, and received treatment at another hospital the following day. The blood routine examination revealed a white blood cell count of 17.63*10^9/L, neutrophil percentage of 80.3%, hemoglobin level of 140 g/L, and C-reactive protein level of 28.4 mg/L. Chest computed tomography indicated bilateral lung infection, with a negative COVID-19 antigen test result. Treatment included intravenous infusion of ceftriaxone and doxofylline. Medical interview showed that the patient have a history of repetitive ceftriaxone administration and the patient reported a history of allergy to levofloxacin and sulfonamides. During the infusion process, the patient experienced fever of 39.6°C, chills, nausea, and vomiting once, passed loose stools twice, and did not develop a rash. The possibility of doxofylline allergy was considered. Doxofylline was then discontinued and methylprednisolone 40 mg as anti-allergy treatment was administered intravenously. Following medication, the chills, nausea and vomiting improved while symptoms of sore throat and cough persisted. Subsequently, the patient exhibited dark-colored urine, resembling soy sauce, on two occasions. Laboratory examination showed blood glucose 24.07 mmol/L, urinary ketone body (++), total bilirubin 32.0 μmol/L, and lactate dehydrogenase (LDH) 345U/L.

Two days later, the patient was admitted to the hospital she visited before with a result of blood routine examination showing hemoglobin level of 112 g/L. Ceftriaxone 2 g qd and methylprednisolone 40 mg qd were continued, with discontinuation of methylprednisolone after 3 days. On the 6th day of hospitalization, the patient presented symptoms of soy sauce-colored urine again after receiving ceftriaxone. Laboratory examination revealed a decrease in Hb to 93 g/L (baseline at the time of first treatment was 140 g/L), LDH 890U/L, and blood sugar level of 10.95 mmol/L. The following day, the patient developed fever, chills, nausea, and vomiting after administration of ceftriaxone. Laboratory examination showed a decrease in Hb to 79 g/L, total bilirubin at 53.8 μmol/L, direct bilirubin at 10.2 μmol/L, indirect bilirubin at 43.6 μmol/L and a positive DAT. Suspecting adverse reactions to ceftriaxone, ceftriaxone was discontinued immediately and symptomatic treatment including a single dose of methylprednisolone 40 mg and insulin were administered. Subsequently, the patient experienced amaurosis, sweating as well as undetectable blood pressure, and was transferred to our hospital’s emergency department for further treatment in the evening on the same day. During the further process of medical history taking, the patient’s family denied a family history of cardiovascular diseases or allergy to ceftriaxone or other cephalosporins.

Upon our hospital’s emergency department visiting, the patient presented with a body temperature of 38.6°C, pulse rate of 88 bpm, respiration rate of 15 times/min, blood pressure of 98/62 mmHg, SPO2 93%, pale palpebral conjunctiva, yellow sclera and skin, clear consciousness but poor mental status. No dry or wet rales were detected in both lungs. Comprehensive laboratory examination revealed white blood cell level at 41.59*10^9/L, red blood cell level at 2.27*10^12/L, Hb level at 68 g/L, reticulocyte count level at 131.0*10^9/L, reticulocyte percentage was 5.77%, LDH level at 1313U/L, total bilirubin level at 37.7 μmol/L, uncongjugated bilirubin level at 29.9 μmol/L, activated partial thromboplastin time (APTT) 25.0 s and type A/B influenza acid (−). Symptomatic treatment including fluid rehydration and oxygen inhalation was administered while the patient remained in the emergency room under critical care observation. At 5a.m. the following day, the patient experienced heart palpitations, sweating and a blood pressure reading of 82/48 mmHg which improved after receiving additional fluid rehydration treatment before being transferred to the ward for further management. The laboratory examination was completed in the ward, which showed that the red blood cell count was 2.09*10^12/L^, Hb 60 g/L, reticulocyte count was 292.1*10^9/L^, reticulocyte percentage was 14.25%, glucose was 11.95 mmol/L, urinary ketone (+), LDH 611U/L, D-dimer 2955 ng/mL, and APTT 21.7 s. There were also the results showing that the plasma free hemoglobin was 1165 mg/L, the haptoglobin was 5.86 mg/dL, and no schistocyte were found on peripheral blood smear.

**FIGURE 1:**
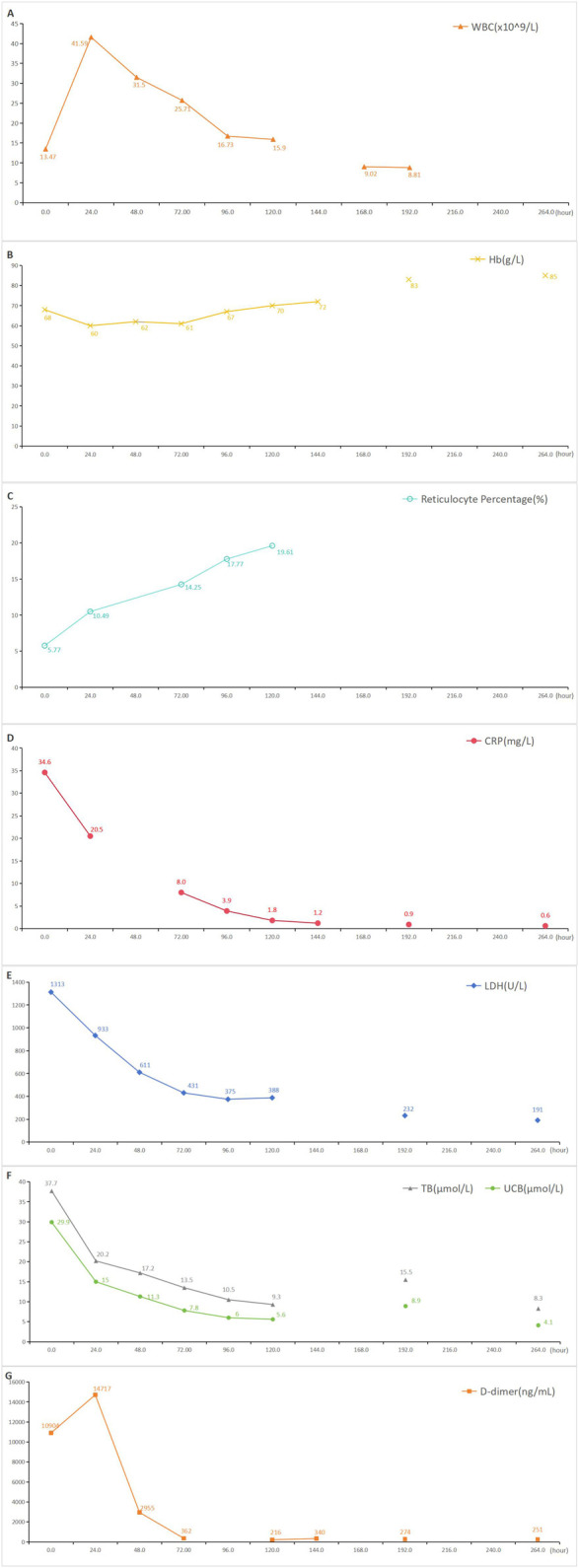
Temporal changes in laboratory tests following hospitalization in our hospital. Blood indices were respectively re-examined before discharge. **(A)**: Temporal changes in white blood cell following ceftriaxone-induced immune hemolysis; **(B)**: Temporal changes in hemoglobin following ceftriaxone-induced immune hemolysis; **(C)**: Temporal changes in reticulocyte percentage following ceftriaxone-induced immune hemolysis; **(D)**: Temporal changes in C-reactive protein following ceftriaxone-induced immune hemolysis; **(E)**: Temporal changes in lactate dehydrogenase following ceftriaxone-induced immune hemolysis; **(F)**: Temporal changes in total bilirubin and unconjugated bilirubin following ceftriaxone-induced immune hemolysis; **(G)**: Temporal changes in D-dimer following ceftriaxone-induced immune hemolysis; WBC: white blood cell, Hb: hemoglobin, CRP: C-reactive protein, LDH: lactate dehydrogenase, TB: total bilirubin, UCB: unconjugated bilirubin.

The patient was suspected of CIHA combined with a respiratory tract infection. The treatment regimen included methylprednisolone 80 mg qd, meropenem antibacterial, enoxparin anticoagulant, insulin intravenous pump hypoglycemic and sodium bicarbonate alkalization. After a hematology department consultation, the laboratory examination was further refined, with DAT (+/−), Immunofixation electrophoresis of blood and urine (−), antinuclear antibodies (−), Epstein-Barr virus DNA (−), alpha fetoprotein and carcinoma embryo antigen (−). CIHA combined with hemolytic crisis and a respiratory tract infection was diagnosed after ruling out other hemolytic causes. Ultrasound examination showed satisfactory cardiac function with the exception of venous thrombosis in both lower limbs. The treatment regimen remained unchanged. Subsequently, the patient’s condition improved and her hemoglobin level gradually recovered. The patient was ultimately discharged after 10 days of hospitalization with a prescription of oral methylprednisolone 40 mg qd, which was demanded to reduced gradually until discontinuation. The one-month follow-up showed a favorable prognosis. A detailed overview of the key laboratory findings and other auxiliary examination during the diagnostic process were provided in Table 1 and temporal changes in laboratory tests following hospitalization in our hospital was shown in Figure 1.

**TABLE 1:** Summary of laboratory investigations and other auxiliary examination.

Investigations	Results	Reference range	Notes
Red blood cell, 10^12/L^	2.27	3.50–5.50	Indicates anemia
White blood cell, 10^9/L^	17.63	4.00–10.00	Suggests infection
Hemoglobin, g/L	68, further decrease to 60	110–170	Indicates severe anemia
Blood gas			Indicates metabolic acidosis
Myocardial enzymes	No abnormality		Suggests no relevant cardiac involvement
Liver function	No abnormality		Suggests no liver injury
Renal function	No abnormality		Suggests no renal injury
Blood glucose, mmol/L	24.07	3.9–6.1	Suggests stress hyperglycemia or poor control of blood glucose
Urinary ketone	(++)	(−)	Implies ketoacidosis
Lactate dehydrogenase, U/L	345	120–250	Indicates hemolytic anemia
Total bilirubin, μmol/L	37.7	3.4–17.1	Indicates hemolytic anemia
Unconjugated bilirubin, μmol/L	29.9	1.7–10.2	Indicates hemolytic anemia
Peripheral blood smear	Normal		Excludes hemolytic anemia associated with abnormal red blood cell morphologies
Reticulocyte count, 10^9/L^	131.1	24–84	Indicates hemolytic anemia
Reticulocyte percentage, %	5.77	0.5–1.5	Indicates hemolytic anemia
Activated partial thromboplastin time, s	25.0, further decrease to 21.7	23.3–32.5	Suggests hypercoagulable state
D-dimer, ng/mL	10,904, further increase to 14,717	0–500	Suggests hypercoagulable state
Direct Antiglobulin Test	Positive	Negative	Indicates immune-mediated hemolysis
Immunofixation electrophoresis of blood and urine	Negative	Negative	Rules out monoclonal gammopathies
Antinuclear antibodies	Negative	Negative	Indicates a lower likelihood of systemic autoimmune disorders
Pathogen tests			Rules out common pathogenic infection-induced immune hemolytic anemia
Epstein-Barr virus DNA	Negative	Negative	
*Mycoplasma pneumoniae* DNA	Negative	Negative	
Serum tumor markers	Negative	Negative	Excludes tumor-induced anemia
Chest computed tomography			Suggests bilateral lung infection
Cardiac ultrasound	No abnormality		Suggests no relevant cardiac involvement
Ultrasound of lower extremities	No abnormality		Excludes thrombus of lower extremity veins

## Discussion

**FIGURE 2:**
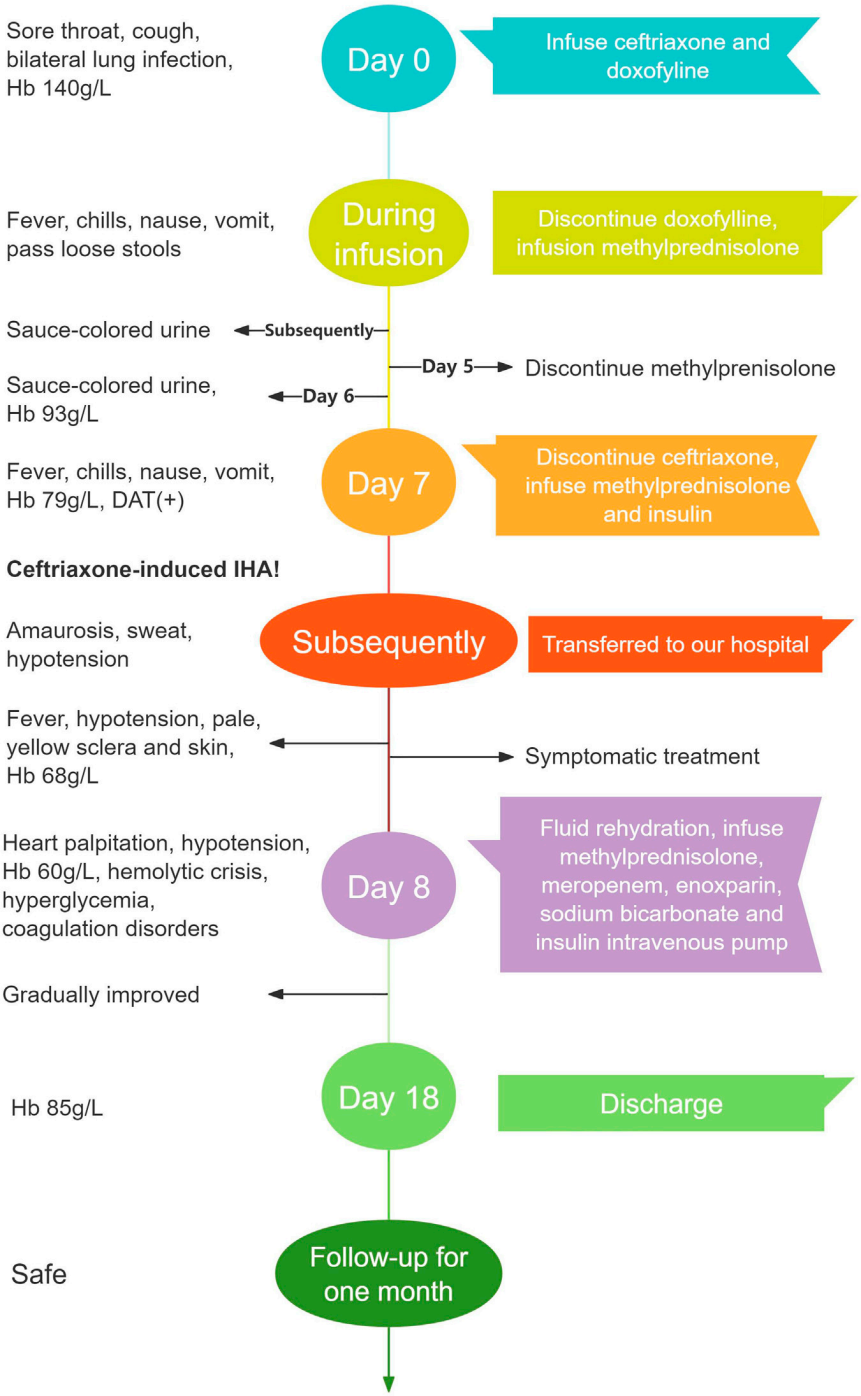
A timeline of the patient’s progress from her first visit to hospital to recovery Hb, hemoglobin; DAT, direct Antiglobulin Test; IHA, immune hemolytic anemia.

This article reported a case of a 54-year-old woman with diabetes mellitus, hypertension and bronchial asthma who developed severe hemolytic anemia, hemodynamic instability and hemolytic crisis after intravenous infusion of ceftriaxone following a respiratory infection. The patient was diagnosed with CIHA after excluding other possible hemolytic etiologies. Timely identification of early hemolysis manifestations, withdrawal of ceftriaxone, and effective treatment led to gradual improvement in the patient’s condition and a good prognosis. Figure 2 showed a timeline with the patient’s symptoms, diagnosis, relevant data and treatment measures during the episode of care.

Nevertheless, this case still offers valuable insights into the use of ceftriaxone. Among the over 130 drugs linked to DIIHA, antibiotics such as cephalosporins and penicillin make up more than 40% (Garratty, 2009; Maquet et al., 2023). Previous reports have suggested that ceftriaxone might be the second most common medication to cause DIIHA (Arndt et al., 2012). The rapid progression of the disease and potential multi-system complications contribute to the higher severity of symptoms and mortality, which might be as high as 50%, in CIHA compared to other drug-induced DIIHA (Dicaro et al., 2024; Mayer et al., 2015; Garratty, 2010). Previous literature has indicated that CIHA has a higher incidence in children and may lead to more severe hemolytic reactions (Leicht et al., 2018).

Possible mechanisms of drug-induced hemolysis include direct erythrocyte toxicity, where drugs bind directly to red blood cells and cause destruction of the erythrocyte membrane, as well as drug-induced immune response leading to immune complex formation mediated by antibodies, complement activation, and subsequent hemolysis (Garratty, 2009; Arndt, 2014). Antibodies related to DIIHA are classified into two classes: drug-dependent antibodies (DDABs) and drug-independent antibodies (DIABs). DDABs, capable of both IgG and IgM types, react with red blood cells and trigger hemolysis only in the presence of drugs, which is the common mechanism that ceftriaxone induces hemolysis. Additionally, DDABs may also attack platelets and cause thrombocytopenia, potentially accounting for the reason why CIHA could complicate disseminated intravascular coagulation (DIC) (Piedra Abusharar et al., 2019). DIABs, similar to red blood cell autoantibodies, can be detected by laboratory examination without the use of disease-causing drugs and independently induce hemolysis. The primary type of DIABs is IgG (Vehapoğlu et al., 2016; Tasch and Gonzalez-Zayaz, 2017).

**FIGURE 3:**
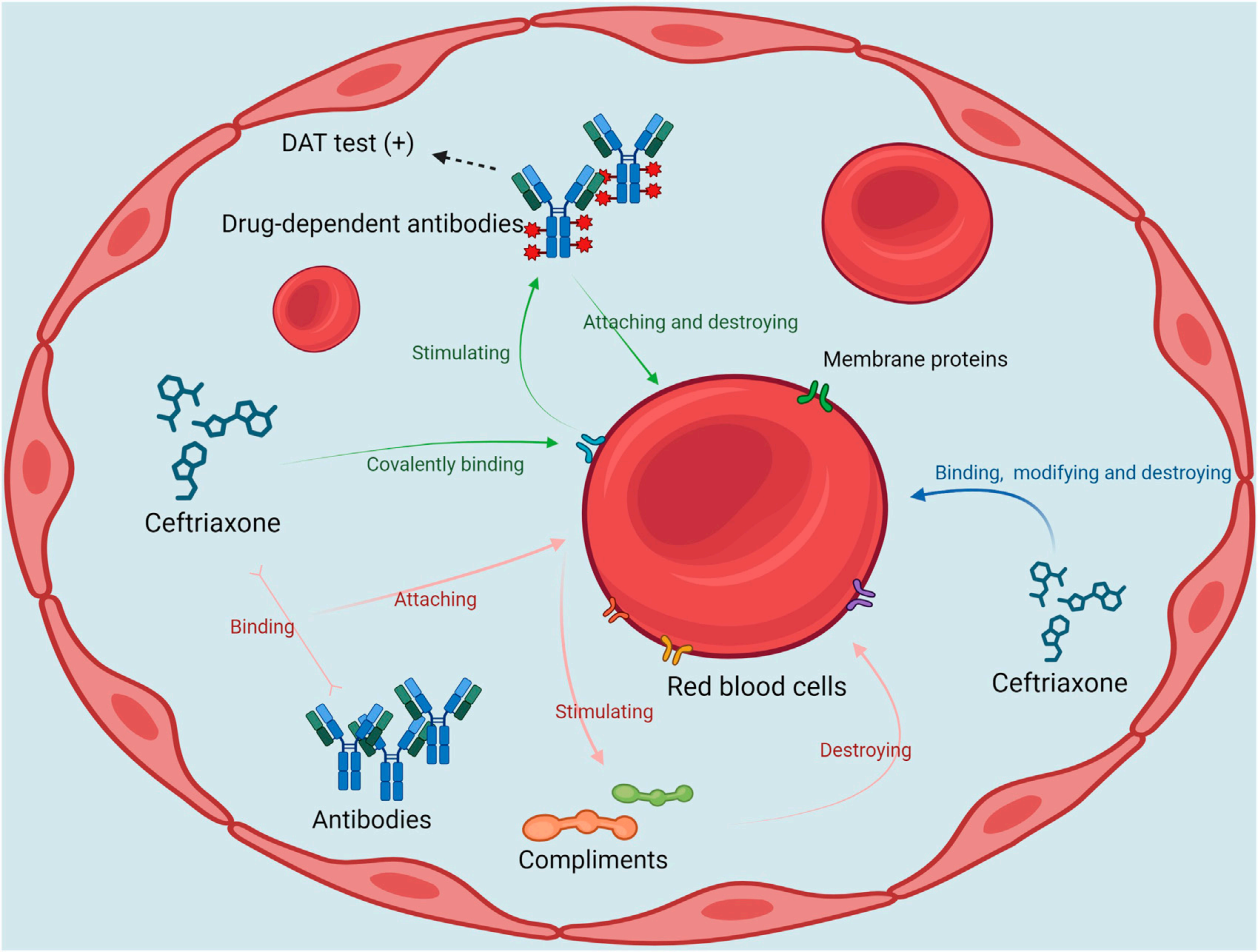
The possible mechanisms of ceftriaxone-induced hemolytic anemia DAT, direct Antiglobulin Test.

It has been reported that ceftriaxone can induce hemolytic anemia through the following three mechanisms. (1) Ceftriaxone covalently binds to proteins on red blood cell membranes as haptens and stimulates the production of high-titer DDABs. These antibodies attach to the red blood cell membrane and cause hemolysis and a positive DAT. (2) Ceftriaxone binds to the antibody to form an immune complex, which then binds to the erythrocyte membrane and further activates the complement, resulting in severe intravascular hemolysis. (3) Ceftriaxone can non-specifically bind to and modify the erythrocyte membrane, and this membrane modification process may lead to the destruction of erythrocyte membrane and thus cause hemolysis. This process is not mediated by antibodies, also known as non-immune protein adsorption (NIPA) (Leicht et al., 2018). The mechanism is analogous to direct erythrocyte toxicity. The three possible mechanisms of CIHA are presented in Figure 3. The longer elimination half-life of ceftriaxone compared with other cephalosporins may elevate the chance of such immune reactions, which may contribute to its increased likelihood of causing DIIHA (Patel et al., 1981).

Medical history and clinical manifestations play an essential role in the diagnosis of CIHA, although laboratory examination remain the primary foundation for current diagnosis of CIHA. A recent record of ceftriaxone usage is essential for the diagnosis of CIHA. It is important to note that previous treatment with ceftriaxone without adverse reactions should not be used as a basis for excluding the diagnosis of CIHA. According to previous literature, 65% of CIHA patients have a history of ceftriaxone exposure, and repeated exposure to ceftriaxone may increase the likelihood of developing CIHA (Neuman et al., 2014; Vehapoğlu et al., 2016; Singh et al., 2020). Due to the pathogenesis of CIHA, it typically takes several days or longer for an immune response and symptoms to develop after initial exposure to ceftriaxone (Renard and Rosselet, 2017), which also explains the delayed onset of severe hemolysis symptoms in this case, occurring several days after ceftriaxone administration. Besides, patients with underlying diseases such as sickle cell disease and HIV have a higher incidence of CIHA (Neuman et al., 2014; Zeng et al., 2020). Previous case reports have underscored the significance of promptly identifying suspected CIHA in patients with underlying disease and a history of ceftriaxone exposure as well (Neuman et al., 2014). In addition to hemolysis-related symptoms such as fatigue, pallor, dark urine, and jaundice, typical manifestations of CIHA include nausea, vomiting, trunk pain, and other complications (Miranda et al., 2022).

Laboratory findings indicative of CIHA include indicators associated with hemolytic anemia, such as decreased hemoglobin, reduced haptoglobin levels, elevated LDH, and hyperbilirubinemia, and immunological indicators suggestive of immune-mediated hemolysis like a positive DAT for C3 and/or IgG/IgM (Arndt, 2014). A positive result in DAT is regarded as the most dependable laboratory indicator (Pierce et al., 2011). The complement hemolysis using human erythrocytes (CHUHE) assay and peptide inhibitor of complement C1 (PIC1) have also been used in previous studies to assist in the confirmation of CIHA (Cunnion et al., 2019). Furthermore, utilizing immunohematological technology to confirm the presence of ceftriaxone-dependent antibodies in patients’ serum samples is also an effective method for diagnosing CIHA. It is advisable to integrate to combine the results of multiple laboratory tests to diagnose CIHA (Arndt, 2014; Salama and Mayer, 2014). In the case we described, the patient developed typical clinical symptoms such as dark urine, nausea and vomiting following administration of ceftriaxone. Her red blood cell count, hemoglobin and haptoglobin levels were significantly reduced, LDH and plasma free hemoglobin were increased, and DAT was positive. Although testing for ceftriaxone-dependent antibodies was not conducted, the findings were supportive of the diagnosis of CIHA.

In addition to hemolysis, a diverse range of serious complications is also a major cause of high mortality rates in patients with CIHA. In previous reports, the incidence of various complications of CIHA may surpass 40%., and prevalent complications encountered in CIHA patients encompass back pain, acute renal failure, cholestasis, diffuse intravascular coagulation, shock, acute respiratory distress syndrome, and hemolytic crisis, among others (Neuman et al., 2014; Goyal et al., 2011). A series of complications including back pain, liver injury, acute renal failure and shock primarily arises from the rapid destruction of RBC and the subsequent release of their contents into the bloodstream during the course of CIHA (Tao et al., 2024; Guarino et al., 2022). Considering that the elimination of ceftriaxone in the human body is dependent on excretion pathways, patients with renal failure or cholestasis may experience more prominent symptoms of hemolysis (Patel et al., 1981). Identifying the potential complications of CIHA is essential for early detection and determining effective treatment strategies. In this case, the patient also experienced complications of hemolytic crisis and hemodynamic instability, which were successfully resolved after appropriate supportive treatment. Furthermore, considering the clear association between DIHC and DIC (Dicaro et al., 2024), the hypercoagulable state indicated by the shortened activated partial thromboplastin time and elevated D-dimer levels in patients may also be attributed to CIHA. Therefore, aggressive anticoagulant therapy with low molecule weight heparin during the course of treatment in this patient is believed to be advantageous.

For CIHA, the most primary and crucial treatment is timely withdrawal of ceftriaxone (Hill et al., 2017). Previous research has indicated that prompt discontinuation of ceftriaxone can significantly decrease mortality in CIHA patients (Tao et al., 2024; Northrop and Agarwal, 2015). The timely identification, intervention, and discontinuation of ceftriaxone for suspected adverse reactions were crucial factors in enabling the patient in this case to avoid serious CIHA complications and achieve a favorable prognosis. Besides, given the potentially life-threatening nature of CIHA, it is imperative for patients to be hospitalized in the intensive care unit for proactive supportive therapy and circulatory assistance as necessary. Timely red blood cell transfusions have demonstrated positive outcomes for patients with CIHA in previous cases (Singh et al., 2020; Boggs et al., 2011), and erythropoietin transfusions with ferrous sulfate, folic acid, and vitamin B12 when necessary may also achieve similar results as red blood cell transfusions (Tasch and Gonzalez-Zayaz, 2017). Platelet and clotting factor transfusions are necessary for patients with DIC or other coagulopathy as well (Dicaro et al., 2024). Since the pathogenesis of CIHA is essentially an immune response, treatment strategies for severe hemolytic reactions may include steroid hormones and immunosuppressants such as cyclophosphamide (Dicaro et al., 2024; Neuman et al., 2014; Wu et al., 2021), despite all that the latter has been infrequently utilized in previous cases. Although the efficacy of steroids for CIHA has been questioned by some researchers (Leicht et al., 2018), in this case, it is believed that the early administration of steroids effectively prevented the patient from further exacerbating the hemolysis. It should be noted, however, that steroid hormones have a physiological effect of increasing blood sugar levels, which may have contributed to the unstable blood sugar levels in this patient with previous diabetes during treatment. This underscores the importance of closely monitoring CIHA patients with underlying conditions when administering treatment. Plasmapheresis is another effective option, especially for patients with acute renal failure (Leicht et al., 2018; Salama, 2009). Plasmapheresis plays a significant role in reducing the levels of specific antibodies and bilirubin in plasma. For patients with CIHA, it can not only restrict the immune response to ceftriaxone-induced hemolysis, but also might exert a protective effect on the disturbance of consciousness caused by excessive unconjugated bilirubin or the damage of red blood cell destruction products to renal function. Using intravenous gammaglobulin (IVIG) is considered to be therapeutic for drug-independent antiboy-mediated intravascular hemolysis, and IVIG infusion has been successfully used in the treatment of CIHA in some cases (Tao et al., 2024; Vehapoğlu et al., 2016; Tasch and Gonzalez-Zayaz, 2017). Although IVIG has mostly been reported as an effective treatment in previous case reports, there are risks of causing liver damage, acute renal failure or even acute hemolysis related to the passive transfer of antibodies (Jiang et al., 2023). Therefore, the administration of IVIG for CIHA patients requires careful consideration. Given the involvement of complement in the pathogenesis of CIHA, complement inhibitors also hold potential therapeutic value (Cunnion et al., 2019).

Moreover, after confirming the diagnosis of CIHA, it is essential to note that the use of ceftriaxone in patients is contraindicated for life. There is no clear consensus on the alternative antibiotic options for patients with a history of CIHA. In consideration of reports of interactions between ceftriaxone and other cephalosporins or drugs including piperacillin (Chaudhry et al., 2019; Lou et al., 2024), it may not be advisable to use beta-lactams as an alternative choice. Quinolones, macrolides, and aminoglycosides could be effective options, and the final choice of dosing regimen should be based on susceptibility results. Given reports of DIIHA caused by antibiotics like levofloxacin (Karunathilaka et al., 2020; Sukhal and Gupta, 2014), physicians should closely monitor the patient’s condition during subsequent treatment and watch for indicators of worsening hemolysis.

## Conclusion

The presented case highlights the necessity of timely recognition and intervention of ceftriaxone-induced hemolytic anemia, which is a rare, but potential life-threatening condition. For patients with suspected CIHA, clinical symptoms and laboratory investigation related to hemolytic anemia as well as Immunological investigation could facilitate a definitive diagnosis. Once CIHA is confirmed, immediately discontinue ceftriaxone is principal and appropriate treatment including vital signs monitoring, circulation support, timely blood transfusion, along with corticosteroids when necessary should be pursued. Immunosuppressant, plasmapheresis and IVIG administration may be used in serious cases.

## Data Availability

The original contributions presented in the study are included in the article or supplementary material, further inquiries can be directed to the corresponding author.

## Abbreviations

CIHA, ceftriaxone-induced hemolytic anemia; CTX, Ceftriaxone; DIIHA, Drug-induced immune hemolytic anemia; DAT, direct anti-human globulin tests; LDH, lactate dehydrogenase; APTT, activated partial thromboplastin time; DDABs, drug-dependent antibodies; DIABs, drug-independent antibodies; DIC, disseminated intravascular coagulation; CHUHE, complement hemolysis using human erythrocytes; PIC1, peptide inhibitor of complement C1.

## References

[B1] Arndt P. A. (2014). Drug-induced immune hemolytic anemia: the last 30 years of changes. Immunohematology 30 (2), 44–54. 10.21307/immunohematology-2019-098 25247622

[B2] Arndt P. A. Leger R. M. Garratty G. (2012). Serologic characteristics of ceftriaxone antibodies in 25 patients with drug-induced immune hemolytic anemia. Transfusion 52 (3), 602–612. 10.1111/j.1537-2995.2011.03321.x 21880048

[B3] Boggs S. R. Cunnion K. M. Raafat R. H. (2011). Ceftriaxone-induced hemolysis in a child with Lyme arthritis: a case for antimicrobial stewardship. Pediatrics 128 (5), e1289–e1292. 10.1542/peds.2010-1570 21969285

[B4] Cannavino C. R. Nemeth A. Korczowski B. Bradley J. S. O'Neal T. Jandourek A. (2016). A randomized, prospective study of pediatric patients with community-acquired pneumonia treated with ceftaroline versus ceftriaxone. Pediatr. Infect. Dis. J. 35 (7), 752–759. 10.1097/INF.0000000000001159 27093162

[B5] Chaudhry S. B. Veve M. P. Wagner J. L. (2019). Cephalosporins: a focus on side chains and β-lactam cross-reactivity. Pharm. (Basel). 7 (3), 103. 10.3390/pharmacy7030103 PMC6789778 31362351

[B6] Cunnion K. M. Feagin L. M. Chicella M. F. Kaszowski C. L. Hair P. S. Price J. (2019). Ceftriaxone-induced immune hemolytic anemia: *in vitro* reversal with peptide inhibitor of complement C1 (PIC1). Case Rep. Hematol. 2019, 4105653. 10.1155/2019/4105653 30838143 PMC6374879

[B7] Dicaro M. V. Chen C. Wang S. Eom A. Y. Wahi-Gururaj S. (2024). Ceftriaxone-induced hemolytic anemia: a rare and fatal reaction. Cureus 16 (5), e59646. 10.7759/cureus.59646 38832173 PMC11146677

[B8] Garratty G. (2009). Drug-induced immune hemolytic anemia. Hematol. Am. Soc. Hematol. Educ. Program 2009, 73–79. 10.1182/asheducation-2009.1.73 20008184

[B9] Garratty G. (2010). Immune hemolytic anemia associated with drug therapy. Blood Rev. 24 (4-5), 143–150. 10.1016/j.blre.2010.06.004 20650555

[B10] Goyal M. Donoghue A. Schwab S. Hasbrouck N. Khojasteh S. Osterhoudt K. (2011). Severe hemolytic crisis after ceftriaxone administration. Pediatr. Emerg. Care 27 (4), 322–323. 10.1097/PEC.0b013e3182131fa8 21467885

[B11] Guarino M. Perna B. Pastorelli A. Bertolazzi P. Caio G. Maritati M. (2022). A case of ceftriaxone-induced liver injury and literature review. Infez. Med. 30 (2), 293–297. 10.53854/liim-3002-16 35693049 PMC9177192

[B12] Hill Q. A. Stamps R. Massey E. Grainger J. D. Provan D. Hill A. (2017). Guidelines on the management of drug-induced immune and secondary autoimmune, haemolytic anaemia. Br. J. Haematol. 177 (2), 208–220. 10.1111/bjh.14654 28369704

[B13] Jiang M. Kimber J. S. Gupta A. Kovoor J. Stretton B. Ravindran J. (2023). Adverse reactions associated with intravenous immunoglobulin administration in the treatment of neurological disorders: a systematic review. Int. Arch. Allergy Immunol. 184 (6), 513–528. 10.1159/000529110 37015212

[B14] Karunathilaka HGCS Chandrasiri D. P. Ranasinghe P. Ratnamalala V. Fernando A. H. N. (2020). Co-amoxiclav induced immune haemolytic anaemia: a case report. Case Rep. Hematol. 2020, 9841097. 10.1155/2020/9841097 32292611 PMC7150724

[B15] Leicht H. B. Weinig E. Mayer B. Viebahn J. Geier A. Rau M. (2018). Ceftriaxone-induced hemolytic anemia with severe renal failure: a case report and review of literature. BMC Pharmacol. Toxicol. 19, 67. 10.1186/s40360-018-0257-7 30359322 PMC6203207

[B16] Lou C. Liu M. Ma T. Yang L. Long D. Li J. (2024). Case report: decreased hemoglobin and multiple organ failure caused by ceftizoxime-induced immune hemolytic anemia in a Chinese patient with malignant rectal cancer. Front. Immunol. 15, 1390082. 10.3389/fimmu.2024.1390082 38756782 PMC11096485

[B17] Maquet J. Lafaurie M. Michel M. Lapeyre-Mestre M. Moulis G. (2023). Drug-induced immune hemolytic anemia: detection of new signals and risk assessment in a nationwide cohort study. Blood Adv. 8 (3), 817–826. 10.1182/bloodadvances.2023009801 PMC10874903 37782770

[B18] Mayer B. Bartolmäs T. Yürek S. Salama A. (2015). Variability of findings in drug-induced immune haemolytic anaemia: experience over 20 Years in a single centre. Transfus. Med. Hemother 42 (5), 333–339. 10.1159/000440673 26696803 PMC4678312

[B19] Miranda S. B. Vilaça J. Ventura Nogueira M. Pontes T. Garcez C. Baptista V. (2022). A near fatal ceftriaxone-induced hemolytic anemia in pediatric age: case report. NCP 9 (2), 1–4. 10.24966/NCP-878X/100101

[B20] Neuman G. Boodhan S. Wurman I. Koren G. Bitnun A. Kirby-Allen M. (2014). Ceftriaxone-induced immune hemolytic anemia. Ann. Pharmacother. 48 (12), 1594–1604. 10.1177/1060028014548310 25163809

[B21] Northrop M. S. Agarwal H. S. (2015). Ceftriaxone-induced hemolytic anemia: case report and review of literature. J. Pediatr. Hematology/Oncology 37 (1), e63–e66. 10.1097/MPH.0000000000000181 24878619

[B22] Patel I. H. Chen S. Parsonnet M. Hackman M. R. Brooks M. A. Konikoff J. (1981). Pharmacokinetics of ceftriaxone in humans. Antimicrob. Agents Chemother. 20 (5), 634–641. 10.1128/aac.20.5.634 6275779 PMC181765

[B23] Piedra Abusharar S. Shah N. Patel R. Jain R. Polimera H. V. (2019). A case of confirmed ceftriaxone-induced immune thrombocytopenia. Cureus 11 (5), e4688. 10.7759/cureus.4688 31338265 PMC6639063

[B24] Pierce A. Nester T. Education Committee of the Academy of Clinical Laboratory Physicians and Scientists (2011). Pathology consultation on drug-induced hemolytic anemia. Am. J. Clin. Pathology 136 (1), 7–12. 10.1309/AJCPBVLJZH6W6RQM 21685026

[B25] Renard D. Rosselet A. (2017). Drug-induced hemolytic anemia: pharmacological aspects. Transfus. Clin. Biol. 24 (3), 110–114. 10.1016/j.tracli.2017.05.013 28648734

[B26] Rolain H. Schwartz Z. Jubrail R. Downes K. J. Hong L. FakhriRavari A. (2024). Meta-analysis on safety of standard vs prolonged infusion of beta-lactams. Int. J. Antimicrob. Agents 64, 107309. 10.1016/j.ijantimicag.2024.107309 39168416

[B27] Salama A. (2009). Drug-induced immune hemolytic anemia. Expert Opin. Drug Saf. 8 (1), 73–79. 10.1517/14740330802577351 19236219

[B28] Salama A. Mayer B. (2014). Diagnostic pitfalls of drug-induced immune hemolytic anemia. Immunohematology 30 (2), 80–84. 10.21307/immunohematology-2019-101 25247617

[B29] Singh A. Singhania N. Sharma A. Sharma N. Samal S. (2020). Ceftriaxone-induced immune hemolytic anemia. Cureus 12, e8660. 10.7759/cureus.8660 32699660 PMC7370697

[B30] Sukhal S. Gupta S. (2014). Drug-induced immune haemolytic anaemia caused by levofloxacin. Singap. Med. J. 55 (8), e136–e138. 10.11622/smedj.2014111 PMC4294100 25189315

[B31] Tao E. Zhou H. Zheng M. Zhao Y. Zhou J. Yuan J. (2024). Ceftriaxone-induced severe hemolytic anemia, renal calculi, and cholecystolithiasis in a 3-year-old child: a case report and literature review. Front. Pharmacol. 15, 1362668. 10.3389/fphar.2024.1362668 38560354 PMC10978768

[B32] Tasch J. Gonzalez-Zayaz P. (2017). Ceftriaxone-induced hemolytic anemia in a jehovah’s witness. Am. J. Case Rep. 18, 431–435. 10.12659/AJCR.903507 28428532 PMC5408558

[B33] Vehapoğlu A. Göknar N. Tuna R. Çakır F. B. (2016). Ceftriaxone-induced hemolytic anemia in a child successfully managed with intravenous immunoglobulin. Turk J. Pediatr. 58 (2), 216–219. 10.24953/turkjped.2016.02.016 27976566

[B34] Wu Y. Wu Y. Ji Y. Liu Y. Wu D. Liang J. (2021). Case report: oral cimetidine administration causes drug-induced immune hemolytic anemia by eliciting the production of cimetidine-dependent antibodies and drug-independent non-specific antibodies. Front. Med. (Lausanne) 8, 723167. 10.3389/fmed.2021.723167 34646843 PMC8504253

[B35] Zeng L. Wang C. Jiang M. Chen K. Zhong H. Chen Z. (2020). Safety of ceftriaxone in paediatrics: a systematic review. Arch. Dis. Child. 105 (10), 981–985. 10.1136/archdischild-2019-317950 32144089 PMC7513262

